# A Rare Case of Anti-HMGCR-Positive Immune-Mediated Necrotizing Myopathy Associated With Statin Use

**DOI:** 10.7759/cureus.101392

**Published:** 2026-01-12

**Authors:** Menkeoma Laura Okoli, Iganiru Chukwuebuka Okoli, Raymond T Anasobi, Abuoma Chisom Okoli, Anulika Francisca Madueke, Ibuchim Chinemerem Okoli

**Affiliations:** 1 Internal Medicine, Laura Okoli MD PLLC, Longview, USA; 2 Internal Medicine, University of Port Harcourt Teaching Hospital, Port Harcourt, NGA; 3 Internal Medicine, Healthcare Research Network, Chicago, USA; 4 Internal Medicine, Harmony Medical and Wellness Center, Abuja, NGA; 5 Internal Medicine, Outreach Women and Children's Hospital, Lagos, NGA; 6 Internal Medicine, All Saints University of St Vincent and Grenadines, Arnos Vale, VCT

**Keywords:** anti-hmgcr antibodies, autoimmune, case report, myopathy, statin

## Abstract

Statin-induced necrotizing autoimmune myopathy (SINAM) is a rare complication of chronic statin use that occurs in individuals aged 50 years and older, with statin exposure ranging from two months to 10 years. While common side effects of statins, such as self-limited myalgia and elevated creatine kinase (CK) levels or rhabdomyolysis, usually resolve with discontinuation, SINAM presents with more severe, persistent, and debilitating symptoms. We describe the case of an elderly man with no prior history of muscle weakness or statin-associated myopathy who presented with dysphagia and progressive weakness in both upper and lower extremities. He was initially diagnosed with statin-induced rhabdomyolysis and received supportive care. However, due to a lack of clinical improvement and based on an expanded literature review, SINAM was suspected. Empiric steroid therapy was initiated, and subsequent anti-3-hydroxy-3-methylglutaryl-coenzyme A reductase (HMGCR) antibody testing returned positive, confirming the diagnosis. This case provides evidence on the use of steroid monotherapy in achieving disease remission. Additionally, it underscores the critical importance of maintaining a high index of suspicion and utilizing evidence-based approaches, especially when common investigative techniques yield no definitive cause. Early recognition and treatment of SINAM are essential to improve patient outcomes.

## Introduction

According to the 224th International Workshop of the European Neuromuscular Centre (ENMC), immune-mediated necrotizing myopathy (IMNM), a clinicopathologic entity of autoimmune myopathies, is classified into three subgroups. These are anti-SRP (signal recognition particle)-positive IMNM, anti-HMGCR (3-hydroxy-3-methylglutaryl-coenzyme A reductase)-positive IMNM, and seronegative IMNM [1]. Statin exposure is strongly associated with the anti-HMGCR-positive subset, and this entity is also referred to as statin-induced necrotizing autoimmune myopathy (SINAM) [1]. SINAM is a rare but serious complication of long-term statin therapy, which occurs in approximately 2-3 cases per 100,000 patients exposed to statins [2-4]. It is typically seen in patients over 50 years of age with a 2-month to 10-year history of statin exposure [2-8].

Statins are commonly prescribed lipid-lowering agents used to treat hyperlipidemia and to prevent cardiovascular events in high-risk individuals [2-10]. The cholesterol-lowering effect of statins is due to the inhibition of HMGCR, a key enzyme in cholesterol synthesis. Although statins are generally well tolerated, they can cause muscle-related side effects such as myalgia, mild creatine kinase (CK) elevation, and rhabdomyolysis, which typically resolve with discontinuation [2, 6, 8]. In contrast, SINAM presents with progressive muscle weakness, elevated CK levels (hyperCKemia), and symptoms that persist despite stopping statins [6, 8, 10]. Severe manifestations of SINAM can include dysphagia and respiratory complications due to bulbar muscle involvement [7, 8, 10].

This report describes the case of a 74-year-old man on atorvastatin diagnosed with statin-induced necrotizing autoimmune myopathy associated with HMGCR autoantibodies. To our knowledge, this is one of the few documented cases in which monotherapy with corticosteroids alone led to complete symptom resolution. This case highlights the value of clinical vigilance and timely intervention when common investigations fail to identify the cause of persistent myopathy in statin users.

## Case presentation

A 74-year-old Hispanic man with a past medical history of hyperlipidemia and type II diabetes presented to the emergency department at CHRISTUS Good Shepherd Hospital, Longview, in September 2024, with complaints of generalized muscle pain and weakness in both upper and lower extremities of four-month duration. The patient reported worsening symptoms that led to multiple ground-level falls and increasing difficulty performing activities of daily living (ADLs). He also described new-onset dysphagia for both solids and liquids lasting over a week. He denied any recent trauma or symptoms of vision changes, fever, rash, or a known family/personal history of autoimmune disease or myopathy. His home medications included atorvastatin (40 mg) and metformin. Additionally, a review of medical records did not show receipt of any vaccinations, particularly COVID, within a year before symptom onset. 

Vital signs on admission were stable with a blood pressure of 125/72, heart rate of 73 bpm, respiratory rate of 18 breaths/min, temperature of 36.8 °C (98.2 °F), body mass index of 26 kg/m^2^, and an oxygen saturation of 94% on room air. Neurological examination revealed intact cranial nerves (II-XII), and normal sensation was elicited for both proximal and distal extremities. However, strength was markedly reduced in all extremities (2/5 for upper extremities and 1/5 for lower extremities). Deep tendon reflexes were present. Cerebellar testing was limited by profound muscle weakness. Physical examination was otherwise unremarkable.

Laboratory tests showed a significantly elevated creatinine kinase (CK) level of 12,867 IU/L (Table 1). Erythrocyte sedimentation rate (ESR) and C-reactive protein (CRP) were within normal limits. Liver enzymes were elevated: aspartate aminotransferase (AST), 100 IU/L, and alanine aminotransferase (ALT), 500 IU/L (Table 1). Complete blood count, blood ethanol level, renal function, and thyroid studies were normal. The patient was diagnosed with statin-induced rhabdomyolysis, made Nil per os, and treated with aggressive hydration and analgesics.

**Table 1:** Laboratory values pre- and post-initiation of steroid therapy

Biomarkers	Pre-steroids	Reference range	Post-steroids
Creatinine kinase (CK)	12,867 IU/L	40-320 IU/L	3,034 IU/L
Aspartate Aminotransferase (AST)	100 IU/L	8-40 IU/L	22 IU/L
Alanine Aminotransferase (ALT)	500 IU/L	7-55 IU/L	49 IU/L
Anti-HMGCR	>200 U/mL	< 20 U/mL	Not assessed

Neurology, gastroenterology, and speech therapy were consulted. Imaging studies, including X-rays of extremities, right upper quadrant ultrasound, and computed tomography (CT) scan of the neck, were all unremarkable. The hepatitis panel, acetylcholine receptor antibody levels, and antinuclear antibodies (ANA) were normal. A modified barium swallow study indicated aspiration risk. An esophagogastroduodenoscopy performed on Day 2 of hospitalization was also unremarkable. Due to ongoing dysphagia, a percutaneous endoscopic gastrostomy (PEG) tube was placed for enteral nutrition. Repeat labs showed a downward trend in CK level (9,886 IU/L) and liver enzymes (AST, 55 IU/L; ALT, 134 IU/L) with normal inflammatory markers. Despite supportive treatment, his weakness did not improve.

Due to persistent symptoms, a broader differential diagnosis was considered. Given his statin history and clinical presentation, SINAM was suspected. A muscle biopsy was attempted but was unsuccessful due to specimen mishandling. HMGCR antibody testing was conducted, and empiric treatment with 60 mg prednisone was started. He was also prescribed calcium and vitamin D for deficiency. CK levels dropped to 3,034 IU/L following steroid initiation, with subsequent normalization of liver enzymes.

He was discharged on hospital Day 12 with home health services and a walker. Follow-up labs showed anti-HMGCR antibody level >200 U/mL (normal <20), confirming the diagnosis of SINAM. The patient was then advised to continue steroids for at least three months and to discontinue statin use permanently. Ezetimibe was initiated for lipid management.

Outcome and follow-up

During initial outpatient follow-up, a few weeks post-discharge, the patient reported progressive improvement in both strength and functionality. Five months later, he noted a marked return of muscle strength in both proximal and distal extremities with downtrending CK levels of < 400 IU/L. He had resumed performing activities of daily living independently and was swallowing without difficulty following the removal of his PEG tube. At that time, he remained on a low-dose prednisone regimen of 5 mg/day, with plans to taper further to 2.5 mg/day. The patient was referred to outpatient physical therapy to support further functional recovery. Ongoing discussions were held about rheumatology referral for long-term monitoring, particularly to prevent relapses during steroid tapering and potential discontinuation. Continuity of care remains a priority to ensure sustained disease remission and optimal quality of life.

## Discussion

This case highlights an uncommon but significant complication of chronic statin use, SINAM. In this case, a patient who was taking a high-intensity statin for over six years presented with complaints of dysphagia and worsening proximal and distal muscle weakness of about a week and four months duration. The initial diagnosis for this patient was statin-induced rhabdomyolysis, which is a commonly recognized side effect. However, symptoms of proximal and distal muscle weakness accompanied by dysphagia raised concern for a more severe pathology.

The underlying mechanism of SINAM, although unclear, may involve the generation of autoantibodies against HMGCR, the enzyme targeted by statins [2, 6, 7, 11-13]. Beyond statins, products containing natural HMGCR inhibitors (such as red yeast rice, Bacopa/Brahmi, and mushrooms containing compounds (shiitake, maitake, reishi)) have also been discussed as potential triggers that activate the autoimmune reaction resulting in the generation of anti-HMGCR antibodies [11, 12, 14]. Additionally, individuals identified to have HLA-DRB1*11 :01 allele are at greater risk of generating these autoantibodies [2, 11-13].

The pathogenic effect of anti-HMGCR antibodies on muscle fiber occurs via binding to the HMGCR protein that results in antibody-mediated injury by complement fixation and membrane attack complex [2, 12-14]. Also, given the persistence of increased HMGCR expression in regenerating muscle membrane following long-term statin use, the immune-mediated damage is typically sustained even after statin discontinuation [13]. This persistence of immune-mediated pathogenesis explains the potential of relapses with treatment tapering as well as the rapid response to immunotherapy [13]. Studies that have compared controls (individuals with inflammatory myopathies) to the disease group (those with SINAM) found that the former do not have anti-HMGCR antibodies [12, 13]. Therefore, anti-HMGCR antibodies are highly specific for individuals with SINAM, correlating with clinical symptoms [12, 13]. This autoimmune component is supported by the presence of markedly elevated creatine kinase (CK) levels, typically exceeding 10,000 IU/L [6, 8-10, 12]. This feature characteristically distinguishes SINAM from other forms of statin-induced myopathies [2, 5-10, 13].

Diagnosis of SINAM can be made either via anti-HMGCR antibody testing [2-10] or through muscle biopsy that shows muscle fiber necrosis with no inflammation [2, 8-10]. Hence, inflammatory markers such as ESR and CRP are usually within normal limits [10]. In our patient, muscle biopsy was inconclusive due to poor sample handling; however, anti-HMGCR antibody testing confirmed the diagnosis. Studies have shown that anti-HMGCR antibody testing has a sensitivity of 94% and specificity of 99%, making it a highly reliable diagnostic tool [2, 5, 8-10]. Given the long turnaround time for results, empiric treatment with corticosteroids was initiated. Remarkably, the patient responded well to monotherapy with prednisone, with significant improvements in muscle strength.

The mainstay therapy for SINAM is immunosuppression that helps to attain disease remission [14]. Currently, there is no formal treatment guideline available for managing patients with SINAM [2, 14]. Reports from limited studies have recommended high-dose corticosteroids as initial therapy [14]. Although steroids are highlighted as initial therapy, monotherapy is usually not sufficient to adequately manage the disease course [2, 14]. Therefore, additional agents such as intravenous immunoglobulin (IVIG), azathioprine, methotrexate, or mycophenolate mofetil are typically added to the regimen [2, 14]. Etanercept, rituximab, cyclosporine, and cyclophosphamide are reserved as third-line agents [13, 14]. Most reported cases in the literature have utilized dual or triple immunosuppressive therapy (e.g., corticosteroids with IVIG, or methotrexate, and rituximab) [2-6, 9, 10, 14]. Also, a study provided evidence on the use of IVIG as monotherapy, with the addition of rituximab in patients who are refractory to dual therapy with second-line agents such as methotrexate [14]. However, our patient achieved full remission with steroid monotherapy, supporting the utility of individualized treatment approaches based on clinical response. Monotherapy with steroids was initially selected as SINAM was still a working diagnosis. Once the diagnosis was confirmed, continued use of steroids was due to healthcare costs and subsequent clinical improvement. While the treatment for other forms of statin-associated myopathy may require a reduction in dose or temporary discontinuation of statins, in the case of SINAM, management involves lifelong avoidance of statins with initiation of immunosuppressants and transition to an alternative lipid-lowering medication [3-9].

Case studies have shown that SINAM is characterized by significantly elevated CK levels of >10,000 IU/L, which decline with initiation of steroid therapy [6-9]. Consistent with these studies, our patient’s CK levels were in the 12,000s on admission and declined to the 3000s with initial therapy (Table 1). The duration of treatment and approach to choice of therapy appear to correlate with clinical improvement [2, 14]. Also, monitoring CK and anti-HMGCR antibody levels can guide therapeutic efficacy and disease resolution [5, 8, 10], as studies have shown that the level of anti-HMGCR antibodies correlates with elevated CK levels, a predictor of increased disease activity [5, 8, 9, 12, 13]. Importantly, this case also emphasizes the value of maintaining a high index of suspicion when common diagnostic approaches fail. Anchoring on common conditions without considering rare but treatable causes can lead to missed diagnoses and delayed care. SINAM, though rare, should be part of the differential diagnoses in patients with progressive muscle weakness, hyperCKemia, and a history of statin use.

## Conclusions

This case provides evidence on the use of steroid monotherapy in achieving disease remission. Also, it underscores the critical importance of maintaining a high index of suspicion and utilizing evidence-based approaches, especially when common investigative techniques yield no definitive cause. Early recognition and treatment of SINAM are essential to improve patient outcomes. Accurate diagnosis in this case led to significant clinical improvement, including resolution of dysphagia, feeding tube removal, a return to functional independence, and permanent discontinuation of the offending agent.
